# Human rights, health and the state in Bangladesh

**DOI:** 10.1186/1472-698X-6-4

**Published:** 2006-04-12

**Authors:** Redwanur M Rahman

**Affiliations:** 1 School of Public Health, La Trobe University, Bundoora, Vic-3086, Australia; 2 Department of Political Studies & Public Administration, Shahjalal University of Science & Technology, Sylhet-3114, Bangladesh

## Abstract

**Background:**

This paper broadly discusses the role of the State of Bangladesh in the context of the health system and human rights. The interrelation between human rights, health and development are well documented. The recognition of health as a fundamental right by WHO and subsequent approval of health as an instrument of welfare by the Universal Declaration of Human Rights (UDHR) and the International Covenant on Social, Economic and Cultural Rights (ICSECR) further enhances the idea. Moreover, human rights are also recognized as an expedient of human development. The state is entrusted to realize the rights enunciated in the ICSECR.

**Discussion:**

In exploring the relationship of the human rights and health situation in Bangladesh, it is argued, in this paper, that the constitution and major policy documents of the Bangladesh government have recognized the health rights and development. Bangladesh has ratified most of the international treaties and covenants including ICCPR, ICESCR; and a signatory of international declarations including Alma-Ata, ICPD, Beijing declarations, and Millennium Development Goals. However the implementation of government policies and plans in the development of health institutions, human resources, accessibility and availability, resource distribution, rural-urban disparity, the male-female gap has put the health system in a dismal state. Neither the right to health nor the right to development has been established in the development of health system or in providing health care.

**Summary:**

The development and service pattern of the health system have negative correlation with human rights and contributed to the underdevelopment of Bangladesh. The government should take comprehensive approach in prioritizing the health rights of the citizens and progressive realization of these rights.

## Background

This paper broadly discusses the role of State in the context of human rights and the health system of Bangladesh. Section I conceptualizes the interrelation between human rights, health and development. Section II analyses the health system development in Bangladesh. Section III discusses how the health system development has not succeeded in progressive realization of health aspect of human rights and further contribution to under-development of Bangladesh. It also gives directions on what should be the government priority to uplift health rights in the context of Bangladesh.

The constitution of World Health Organization (WHO) focused upon relationships between health and human rights. It stated that "the enjoyment of the highest attainable standard of health is one of the fundamental rights of every human being without distinction of race, religion, political belief, social and economic condition" [1]. The Declaration of Alma-Ata [2] of "health for all" in 1978 and the Ottawa Charter for Health Promotion [3] in 1986 further embraced the need for social and economic inputs to improve the health of the population. The Universal Declaration of Human Rights (UDHR) of 1948 and the International Covenant on Economic, Social and Cultural Rights (ICESCR) in 1966 further enunciate the appropriateness of health and human rights for the well being of individuals and the family [4,5]. So there is profound affiliation between human rights and health.

### From civil & political rights to social, economic rights and development

The human rights have recognized not only the civil and political rights but also the social, economic and cultural rights by giving importance to the latter through articulating and prioritizing rights to health, education, housing, and employment. Moreover, the fundamental tenet of the human rights is that every individual's dignity should be protected being a human. This dignity merely means not only political liberty but also a guarantee of economic subsistence, cultural freedom and the provision of social services [6,7]. In this context, the ICESCR deals with the State's obligation to create affirmative conditions to facilitate human well-being. The entitlements of these services are clearly mentioned in UDHR Articles 21 and 25; and ICESCR articles 2, 6, 9, 11, 12, 13, 14. Article 25 of UDHR indicates that "every one has the right to a standard of living adequate for the health and well-being of himself and of his family, including food, clothing, housing, medical care and necessary social services, and the right to security in the event of unemployment, sickness, disability, widowhood, old age or other lack of livelihood in circumstances beyond his control" [[4], UDHR Article 25]. At the same time, ICESCR further defines these rights by obligating the state to undertake steps to maximize available resources with a view to achieving progressive realization of these rights [[5], ICSER Article 2]. Moreover, the state should not only give importance towards economic, social and cultural rights but the same amount of attention and importance also should be given to civil and political rights. In addition, the United Nations declaration on right to development in 1986 further advanced the 'idea' that rights should be renamed as an instrument of development. It is argued that civil and political rights should be fought for on the socio-economic and cultural fronts, and this has been conceptualized as the 'right to development' [8-11]. Since then, 'rights' have been focused on as an agent of development that has been affirmed and reaffirmed in different fora. The World Conference on Human rights in 1993 describes right to development as universal and inalienable [12]. So it can be said that human rights means all aspects of rights which is further linked to development.

### State and social rights

The ICSCER gives obligation to the state authority to ensure social rights. In a particular socio-political, historical, cultural and economic environment; society, social structure, political process and the power relations try to alleviate human miseries. Moreover, the state structure can facilitate and guarantee the social human rights to every individual in accessing to essential levels of social services.

There is a common understanding that it is the responsibility of the State to facilitate social rights. Though UDHR did not create any legal binding or obligation on state parties, state parties viewed it as obligation to maintain basic and minimum international human rights standards. In 1966, International Covenants on Civil and Political Rights (ICCPR) and ICESCR imposed binding obligations on state parties. The ICESCR emphasizes that state parties require positive steps towards progressive achievement of the full realization of rights, which is incorporated in the covenant. It further imposes that state parties have an obligation to ensure the satisfaction of at least the minimum level of each right [13]. The *Limburg Principles *adopted in 1986 clarified that social rights could be guaranteed and implemented in different socio-political and economic settings [14]. The *Principles *favour ensuring and respecting for minimum subsistence rights for all, regardless of economic level development. The state should utilize its legal, administrative, economic, social, educational and related means to materialize the obligations of the covenant. So the state is entrusted to facilitate social rights to every citizen of a country. Hence the responsibility lies with the state to ensure and to guarantee individual's access to requisite resources to live in a dignified way. These requisite resources may include economic, social, cultural, civil and political rights. The guarantee of these social goods is necessary preconditions of the enjoyment of all human rights and allows individuals to participate fully in all other areas of their lives [7]. The intervention of the state is required for mobilizing of resources and expenditure for the fulfillment of social rights [7,15]. Moreover, the implementation of social rights also depends on state's active involvement and participation in policy-making, policy implementation, budgetary allocation and priorities to ensure that every individual will be able to receive his or her entitlements. So every state should take appropriate action to initiate the 'right to development' in every aspect to ensure equality of opportunity for all in their access to basic resources such as health, education, food, housing, employment, and fair distribution of income, thus recognizing social rights.

### State and health rights

The role of state in the provision of health rights further ensures states' obligation to provide minimum care to every individual. The right to health imposes three obligations on states, i) to respect ii) to protect and iii) to fulfil and to promote the enjoyment of the right to health [16]. The state should respect the health rights of every individual through protecting them from illness and diseases, facilitating and providing minimum basic services and health promotion. If there is a natural calamity, or sudden out-break of diseases in part or in the whole country, the government should take necessary measures to protect the citizens. The state should maintain minimum basic conditions of the institutional facilities to provide health services to every individual irrespective of caste, class, creed, religion, and geographical location. Providing basic services also respects the citizens' right to health care. The state should also need to promote health rights to individuals. The promotion of health indicates not only health services but also providing necessary services to ensure safe food, hygienic shelter, potable water, sanitation and drugs. In addition, health promotion also includes legislative, financial, societal, and organizational change to promote healthy life styles for the well-being of the citizens [3]. So the state is responsible for health promotional activities.

The state needs to facilitate the availability, accessibility, and to maintain acceptability, quality and standard in the provision of health care. The presence of basic facilities for health and health services are drinking water, sanitation, hospitals, clinics, trained health personnel, and essential drugs. Accessible denotes such a health service that is easily accessible without any barrier. The barrier includes financial, geographical, religious, class and caste. Maintaining standards requires acceptable criteria and scientific and medically tested procedures to ensure quality [16]. The state needs to maintain standard of quality of care in the provision of health services.

### State and development

The declaration of the 'right to development' by the United Nations in 1986 gives new impetus to the state to play a new role in advancing development with a new vision. In pursuing development goals, the state should take appropriate action to initiate the 'right to development' in every aspect and must ensure equal opportunity for all. It should not make barriers to the access to basic resources such as health, education, food, housing, employment, and fair distribution of income. It is the State's responsibility to facilitate individual access to requisite resources to live in a dignified way. These requisite resources may include economic, social, cultural, civil and political rights. However, it is observed that in many cases, the state's nature does not allow for enjoyment of basic human rights by providing and ensuring availability and accessibility to health care, sufficient food, basic education, employment and adequate livelihood [17-19]. All states should explore viable options to fulfill the goal of human rights, which lead to development. Moreover, the state's role in alleviating and mitigating social disparity, misery and deprivation may bring social change towards development. The State's provision of guarantees for social rights should help the poorer, weaker and disadvantaged groups to get their due share in society, which also leads to 'development'.

### Human rights, health, and development: their interrelationship

**Figure 1:**
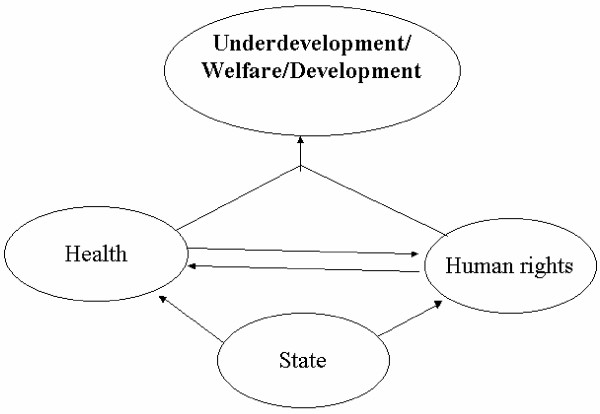
The interrelation between health, human rights and development.

An understanding about the social right to health focused upon a new dimension about the relationship between human rights and health. It gives an idea to maximize the benefit of social good irrespective of social strata, class, race, sex, and religion. Health is universally recognized as an important aspect of human development. Without the development of health, overall development is not possible. On the other hand, human rights are also a part of development. So the interface between human rights and health is towards the development of human welfare (Figure 1). We have mentioned how the declarations of UDHR and ICESCR have integrated health as a part of human rights. We also mentioned that the constitution of World Health Organization (WHO) also recognizes that the highest attainable standard of health is a fundamental right of every human being. Article 12 of ICESCR delineates specific goals to attain better health provision which includes 'right to the highest attainable standard of physical and mental health', development of child health, improvement of environmental and industrial hygiene, prevention and treatment of disease, reduction of infant mortality rate, and the creation of health facilities which are easily available and accessible for the sick [[5], ICSECR Article 12]. Moreover, WHO defined health as "a state of complete physical, mental and social well-being and not merely the absence of disease or infirmity" [1]. The Alma-Ata declaration of 1978 further enhanced health dimension as treating health as a "social goal whose realization requires the action of many other social and economic sectors in addition to the health sector" [2]. The WHO definition gives importance of health promotion as "the process of enabling people to increase control over and to improve their health". So the modern concept of health includes not only health care but also embraces the broader societal dimension to include population well being. So the vision of human rights and health is well documented to ensure human welfare [20-22]. The international declaration of health rights also proclaims "enjoyment of the highest attainable standard of health is one of the fundamental rights of every human being" and health "is not a privilege reserved for those with power, money and social standing" [23]. The General Comment 14 further states that "health is a fundamental human right indispensable for the exercise of other human rights. Every human being is entitled to the enjoyment of the highest attainable standard of health conducive to living a life in dignity" [13]. The inter-linking between human rights and health will not only help to improve the development of health status but human development in general and also embrace equity, solidarity and social justice [24]. Hence human rights and health are inextricably linked in advancing human welfare and development.

## Discussion

### State, human rights and health system in Bangladesh

Bangladesh is located in the north-eastern part of South Asia. Bangladesh became independent from Pakistan in 1971 after a fierce civil war. Administratively, the country is divided into six divisions, 64 districts, 472 *upazillas *(sub-districts), 496 *thanas *(police stations) and 4,451 unions which serve as the basic unit of administration. According to the United Nations Children's Emergency Fund, the estimated population in Bangladesh was 140.36 million in 2001. The per capita gross national product was US$ 460 in 2004. Currently the literacy rate is 65 per cent while 49.8 per cent live below the poverty line and about 30 per cent live on US$ 1 a day. The per capita total expenditure on health was US$ 14 in 1999 [25]. According to a household expenditure survey of 1995–96, the top five per cent of the households in the urban areas have an income of US$ 641 and in the rural areas, it is US$ 240 [26]. Another top 20 per cent of households in the urban areas have very good income. The Bangladesh Bank, Central Bank of Bangladesh, disclosed that 45,000 people have deposits over US$ 1,61,290.32 each and 500 have over US$ 8,06,451.61[27]. In the Bangladesh Parliament, on the basis of professional categories of the parliamentarians, there were about 23 per cent representation from business and industrialists groups in 1973 but their representation has increased to 59 per cent in 1991 [28] and 74 per cent in 1996 and 81 per cent in 2001 [29]. It is revealed from Bangladesh political scenario that moneyed men are in centre of politics and they influence in decision-making process. Thus it indicates that political and economic powers are concentrated on a few people who control and maintain public policies to ensure their part in gaining resources and accessing various public sector facilities.

### Structure of public health care sector

The Ministry of Health and Family Welfare (MOHFW) is the largest institutional health care provider in Bangladesh. Its services range from primary to more complex treatments; its structure is centralized. All decisions regarding the development of personnel and facilities, the allocation of resources and the formulation of policy are made at the central level by the MOHFW.

Specialized institutions operate at the national level. These institutions provide a wide range of services, including cardiology, cardiac surgery, dentistry, endrocrinology, medicine, nephrology, neuro-surgery, oncology, opthalmology, orthopaedics and psychiatry. They offer both inpatient and outpatient services. Ideally, these facilities provide 'follow-up' care for patients referred by various medical college hospitals and other hospitals. However, the referral system works on a very limited scale in Bangladesh as referral *process *has not been fully developed. Their bed capacity varies from 50 to 600, while there are 3,325 beds in national-level hospitals [30,31] (see Table 1).

**Table 1:** Pattern of Health Care Growth in Bangladesh, 1973 and 2003

**Type of Facility**	**1973**	**1978**	**1983**	**1988**	**1993**	**1998**	**2003**
Total Hospitals	308	424	724	875	903	1,273	1,464
Government hospitals	NA	388	560	608	611	647	654
Private Hospitals	NA	36	164	267	292	626	810
District Hospitals	13	37	43	59	57	59	59
*Upazilla *Health Complexes	160	253	319	352	372	402	417
**Number of Beds**							
Total Beds	12,311	19,538	25,057	33,334	35,280	41,514	44,275
Number of Beds in Public Sector	10,449	16,853	20,286	26,871	27,637	30,143	32,615
Number of Beds in Private Sector	1,862	2,685	4,771	6,463	7,643	11,371	11,660
**Teaching Facility**							
Total Medical Colleges	8	8	9	9	16	19	33
Private Medical Colleges	NA	NA	NA	NA	3	6	20
Postgraduate Institutes	1	3	6	6	6	6	6
**Personnel**							
Registered Doctors	5,001	7,035	11,496	18,030	21,004	29,613	36,553
Registered Nurses	765	2,011	5,164	7,390	9,655	16,104	19,066
Registered Midwives	764	1,041	3,424	6,556	7,713	14,312	16,553

Note: NA indicates not available Source:

[30, 31, 41, 66–68]

At the regional level, medical college hospitals provide a range of specialized laboratory facilities for the treatment of complicated cases. These hospitals are required to receive cases referred by the *Upazilla *Health Complexes (UHC) and district hospitals. There are 13 public medical college hospitals with bed capacities of 250 to 1,400, with a total bed capacity of 8,000 [30].

At the district level, there are 59 hospitals (as shown in Table 1). These hospitals are expected to receive cases referred by UHCs. They provide specialist, laboratory and diagnostic services. District-level hospitals have bed capacities ranging from 50 to 250 beds, with a total bed capacity of 5,295 [30].

At the *upazilla *(sub-district) level, there are 417 UHCs (as shown in Table 1), each with a minimum bed capacity of 31 to 50. *Upazilla *Health Complexes are designed to provide primary health care services and to function as referral institutions for Union Sub-Centres (USC) or Union Health and Family Welfare Centres (UHFWC). There are approximately 13,000 beds at the *upazilla *level [32].

There are a total of 4,400 USCs and UHFWCs. These are smallest and most peripheral health, family planning and mother and child health care units, providing only outpatient services for simple injuries and ailments. They have no surgical or bed facilities. According to the HPSP, the government planned to build one community clinics *per *6,000 people. However, the government has built 11,000 community clinics but they are not yet operating [33]. At the ward level (with a population of 6,000–7,000 and comprises two-three villages), health workers provide 'doorstep' services. A health worker visits each household once every four to eight weeks to provide domiciliary services.

### Government policy position

Since the emergence of Bangladesh, the government has given utmost priority to ensure human rights and dignity of the population. Constitutional provisions have been made to protect, respect and to promote individual as well as collective rights in the society. Moreover, Bangladesh is a signatory to most of the international treaties, declarations and ratified covenants to ensure the 'right to development' as a means of promotion of human rights. It also ratified the ICCPR and ICESCR [34].

The country recognizes its obligation to protect and promote human rights. Civil and political rights are recognized in the constitution as fundamental rights. Article 11 of the Bangladesh constitution states "the Republic shall be a democracy in which fundamental human rights and freedoms and respect for the dignity and worth of the human person shall be guaranteed" [[35], Article 11]. Even social, cultural and economic rights are included. The constitution mandates that "it shall be a fundamental responsibility of the state to attain, through planned economic growth, a constant increase in productive forces and a steady improvement in the material and cultural standard of living of the people with a view to securing to its citizens (a) the provision of the basic necessities to life, including food, clothing, shelter, education and medical care" [[35], Article 15]. Article 16 of the constitution also mentions that the state shall adopt effective measures to reduce disparity in health care progressively. Article 18.1 also depicts that the state shall foster rising levels of nutrition and the improvement of public health measures and Article 19 gives importance towards reducing inequality. Bangladesh has given high priority to the development of social sector including health and education with high level of political support. The constitutional provision also guaranteed employment with reasonable wage, right to social security, and quality of life [[35], Article 15, 16, 18, & 19]. The constitutional provisions are made to protect, promote and respect health care as a constituent of human rights in Bangladesh.

The Government of Bangladesh (GOB) has invested substantially in building institution and strengthening of the health care system. It has accepted the goal and reiterated firm political and social commitment to achieve the Primary Health Care (PHC) strategy declared in Alma-Ata in 1978. Moreover, Bangladesh is a signatory of International Conference on Population and Development, Womens' Conference in Beijing and most recently the UN special session on Children's Rights and other important international declarations. Bangladesh signed United Nations Millennium Development Goals which emphasized improvement of maternal health, stemming the spread of HIV/AIDS, malaria and other diseases. In 1998, the Ministry of Health and Family Welfare formulated Health and Population Sector Strategy (HPSS) [36]. It intends to increase quality, equity, efficiency and integration of health and family planning services. As a part of the implementation process, the government has also formulated a Health and Population Sector Programme (HPSP) [37]. It prioritized an essential service package for primary care. Even for the first time, the government announced National Health Policy in 2000. The preamble of the policy document states that the provision of health for the people is the constitutional obligation of the state, [38]. The policy plan on Health, Nutrition, and Population Sector Programme (HNPSP) of 2004 also recognizes health care as basic rights of every citizen [39]. The HNPSP emphasized the necessity for the improvement of maternal and child health care, and facilitating essential service package. Recently prepared Poverty Reduction Strategy Paper (PRSP) gives importance on the enhancement of health [40]. All the plans, programmes and documents have amply demonstrated the government's commitment to making essential health care accessible to every individual and community.

### Status of health indicators and institutions

The Government has given efforts to develop a network of health care systems from village level to national level to cater to the masses. In response, different types of hospitals and other ancillary services have emerged at different levels i.e union level to national level. There were 417 *Upazilla *Health Complexes (UHC), 4,400 Union Sub-Centers or Union Health and Family Welfare Centers (USC/UHFWC), and 1,464 hospitals [41] (see Table 1). There are improvements of morbidity, mortality, fertility and life expectancy at birth (LEB) (see Table 2). But these achievements are much lower than those of the neighbouring countries and far below the regional and global averages [9,42].

**Table 2:** Basic Health Indicators of Bangladesh in 1973 and 2001

**Indicator**	**1973**	**2001**
Infant Mortality Rate (IMR)	140/1000	51/1000
Maternal Mortality Rate (MMR)	30/1000	3.5/1000
Crude Birth Rate (CBR)	47/1000	19.9/1000
Crude Death Rate (CDR)	17/1000	4.8/1000
Life Expectancy at Birth (LEB)	45 years	62 years
Doctor/population ratio	1:6250	1:4105
Doctor Nurse ratio	-	2:1.7
Bed population ratio	-	1:3154
Immunization coverage under one year	-	80 per cent
Proportion of one year old children immunized against measles	-	64 per cent
Total population covered by essential health care	-	42 per cent
Proportion of diarrhoea control	-	70 per cent
Delivery assisted by a trained person	-	14 per cent
Prevalence of Low Birth Weight	-	25 per cent
Prevalence of Child Malnourishment	-	48 per cent

Sources: [43, 69, 70]

The public policies of Bangladesh are directed towards development to alleviate people's misery and protect their rights. But the poor implementation of these policies has given little benefit to the poorer and disadvantaged groups. Different policy documents of the government of Bangladesh reveal that the government target was to achieve 50 per cent deliveries by a trained person by 1995, but the shocking history is that by 1997, it was only 14 per cent. The first five year plan aimed at establishing one *Upazilla *Health Complex in each *upazilla *and one Health Center in each union but, till today (2003), more than 55 *upazilla a*nd more than 200 unions do not have any health facilities. The government had planned to provide essential health care to 80 per cent of the population by 1995 but they had only provided about 45 per cent [43-46]. Moreover, the government has generally emphasized the expansion of the physical infrastructure, like the building of hospitals. Since independence, government has built 654 hospitals, and 4,400 USCs/UHFWCs, 72 dispensaries, 96 maternity clinics, and 32,615 beds. But most of these facilities lack laboratory facilities, required manpower, equipment and furniture. A survey of 16 UHCs, 12 Rural Dispensaries, and 100 USCs shows that 63 per cent had inadequate physical facilities, 60 per cent had inadequate personnel, and 80 per cent faced shortage of supplies or vaccines [47]. While the government counts the number of buildings constructed in assessing its performance in the sector, the creation of these facilities has not ensured services to the population irrespective of place and class i.e. rich and poor. The media reports a very dismal picture of public health facilities in Bangladesh.

This Table 3 shows that the privileged patients from the richest quintile are admitted for in-patient care five times more than the patients from the poorest quintile. The urban patients are more than twice advantaged over the rural patients and the male patients are more likely to get adequate and quality treatment than the female patients. The lowest 20 per cent receive only 16 per cent while the highest 20 per cent receive 26 per cent of all health expenditure [48]. It shows that people from upper echelon is more benefited than lower echelon.

**Table 3:** Use of Public Facilities by Level (in percentage)

**Income Group**	**Hospital Visits**	**UHC Visits**	**Union level facility visits**
Poorest quintile	13	23	26
Second quintile	17	20	19
Third quintile	25	23	21
Fourth quintile	23	20	17
Richest quintile	22	14	17
**Residence**			
Rural (82 per cent)	65	89	83
Urban (18 per cent)	35	11	17
**Gender**			
Male (51.3 per cent)	48	53	55
Female (48.7 per cent)	52	47	45

Source: [37].

### Manpower situation: specialists and urban biases

In Bangladesh public health care is facing a shortage of personnel. Approximately 12,000 physicians work in the public sector [31]. Chaudhury and Hammer report that more than 26 per cent of positions in all categories of health personnel are vacant in public health facilities. The vacancy rate for doctors is 41 per cent. This figure represents more than 2,000 public physician positions [49]. The vacancy rate is higher in rural and poor regions. Moreover, the public health services are gradually tending to have more specialists, rather than mid level health personnel including paramedics, nurses and auxiliary health personnel. The following shows that though there is a gradual improvement of doctor and other health personnel ratio, it still is much lower than global averages [9]. The developed countries have more mid level health personnel compared to doctors but developing countries like Bangladesh have the opposite picture [9]. It is observed that the state has produced more specialist than mid-level health personnel. During 1973, there were 5,570 doctors including dentists. The number had increased to 17,560 in 1985 and further to 27,646 in 1997. But the number of mid-level health personnel (i.e. Nurses, Medical Assistants, Pharmacists, Radiographers, Laboratory Technician, Sanitary Inspector and Dental Technician) was 3,665, 18,865 and 26,715 respectively, in the same period [43-46,50,51]. Since independence, the government has invested more on medical colleges or development of specialized medical institutions but little attention has given to develop paramedical and nursing institutions. This leads to development of more physicians than paramedics, nurses and other auxiliary personnel.

In the early 1970s, there was only one postgraduate, eight graduate and one paramedical institute. Currently, there are six post graduate institutes, 14 medical colleges, three dental colleges, as against two para-medical institutes, five medical training schools, one college of nursing and 38 nursing institutes in the public sector. Every year 1,250 postgraduate and graduate doctors are in the profession but the number of paramedics is less than 200 and that of nurses about 900 [44,45,50,51]. The turn-out of health technologists, paramedics and nurses is below the national requirement. The revenue expenses for medical colleges and other training schools reveal that from 1985–86 to 1989–90, the share of the medical colleges was 3.3 per cent as against 1.2 per cent share of the training schools. From 1990–91 to 1994–95, the share was the same for both the sectors [52]. The manpower development plan and the resource allocations indicate the high preference for fully-fledged and specialist doctors. These circumstances show that paramedics and nurses get very little attention.

Furthermore, the health system is urban biased in facility development and resource distribution. There were 15,706 beds available in the urban areas and the share of the rural areas was 11,297 in 1990 [53] and the comparative figures in 1998 were 14,037 and 12,292 respectively [46,51]. It is also seen that all the specialized and super-specialized hospitals and 14 medical colleges, are located in the city centres only [28].

The manpower distribution is also more urban oriented. Generally, physicians prefer to locate their practice in the urban areas where they get better income opportunities, better living facilities and other socio-cultural services. There is shortage of doctors in the union and *upazilla *level health centres. The government is not able to provide even a graduate doctor in all the union level health facilities but there is an over concentration of health personnel in the urban area. It is observed that there were 359 doctors at Institute of Postgraduate Medicine and Research (presently Medical University), 384 in Dhaka medical college, 253 in Salimullah Medical College in 1990 [54].

Absenteeism is another problem which is common in the public health care system. A background study of the World Bank Report (2004) found that 42 per cent of all categories of health personnel employed in public facilities are usually absent. For physicians the absentee rate at the *upazilla *level is 40 per cent and at the union level 74 per cent [49]. The recent media reports also support that many regional and rural districts and *upazilla *have shortage of doctors and other auxiliary health personnel. Various national daily new papers always reports about shortage of manpower and instrument s in public health facilities at UHC and District level. So it can be said that manpower development and resource distribution has been directed to specialists and urban biases.

### Level of patients care

There has been a decreasing trend of outpatient use at government health facilities. In 1993, there were 23.50 million outpatient attendance in the hospitals that declined to 15.65 million in 1996; for UHCs and for USC, the figures were 24.98 million in 1993, which declined to 17.18 million in 1996 [51]. Another study confirms the same findings. It is found that in 1984, about 20 per cent of the rural patients suffering from acute illnesses were treated in the public sector. This declined to 13 per cent in 1987 and to 12 per cent in 1994 [55]. This declining trend can be attributed to the non-availability of doctors, drugs, and inadequate attention by the doctors, and also distance from home. A study indicates that a minimum of 28.1 per cent mentioned inadequate attention from the doctors, 25.7 per cent talked about the non-availability of drugs, 4.9 per cent about the long waiting time, and 9.2 per cent about the very long distance [56]. It shows that those who go to seek health care from public facilities are directly and indirectly neglected by the functionaries of health facilities. This leads to greater use of private facilities. A World Bank report states that the private sector was used widely: 56 per cent of caesarian; 92 per cent of children with diarrhoea and 75 per cent of "first consultations" were conducted in this sector. The same study reports that, of those who sought health care for illness, 87 per cent of the "urban sample" and 75 of the "rural sample" consulted private providers [57,19,20]. The editorial of an English- language daily newspaper commented that there "has been the erosion of public trust so that fewer and fewer people are turning to the government doctors for medical services" [58].

The Bangabundhu Sheikh Mujib Medical University, the only medical university and the highest level of health facilities in the country, has mostly paying beds. It is difficult for the general population to get services there. There are other specialized and medical college hospitals which do have a good number of free beds. But shocking fact is that the general population get difficulty in getting admission to these facilities. So the influential and the affluent have greater access to the services of these hospitals, whether in the public or the private sector. The poorest and the rural residents receive the least service or benefit from the government facilities.

### Priority on curative care

There have been specific services for preventive and curative care within the public health care system. But the public health services in Bangladesh focus on curative care rather than preventive care. The plans of the government reveal priority on preventive care, but in practice, all efforts are directed towards curative care. In the early 1970s, preventive uni-purpose programmes were launched to control malaria, small pox and other epidemic diseases. In the late 1970s, all the uni-purpose (vertical) programmes were integrated and the preventive efforts were shifted to childhood diseases only through an expanded programme of immunization and health education. In the 1980s, the preventive health programmes became a part of the development programmes for health.

The revenue expenditure (which provide recurring cost) on preventive care was around 8 per cent in the early 1980s and only 0.14 per cent in early the 1990s. But the shares of allocation increased in Annual Development Programmes (ADP) from 13 per cent in the early 1980s to 25 per cent in the early 1990s. In spite of this increasing trend, the total revenue expenses and development allocations together is not more than 8 per cent of the health sector allocation for preventive care [52]. However, three-quarters of morbidity originated from infectious and parasitic diseases. If the government takes appropriate measures to control the above mentioned, only then it is possible to prevent them but lack of appropriate measures like resource allocation for preventive care indicates a relative indifference about preventive care. Hence we see biases for curative care. It indicates that curative care is more admired than preventive care.

### Expenditure pattern

The allocation pattern in the health sector indicates the nature and extent of the government's commitment to health sector's development. The allocation pattern shows that from the first five year plan to the fourth five year plan, the share of allocation to health of the total plan allocation was respectively 3.32 per cent, 3.72 per cent, 2.20 per cent, and 3.05 per cent, and at the same time utilization was 90.47 per cent, 90.79 per cent, 79.63 per cent, and 99 per cent [59]. During the two-year plan holiday period (1995–1997), the allocation was 4.61 per cent of national outlay and utilization was 77.44 per cent [46]. The allocation and utilization pattern shows poor commitment of government for the development of the health sector.

The expenditure pattern also reveals the bias of the government towards the urban residents. The per capita expenditure in the public sector in the urban areas is TK. 118 for in-patient service and TK. 79 for out-patient services. But the corresponding share of the rural areas is TK. 41 and TK. 37, respectively (see Table 4). The amount of total expenditure on the medical personnel also indicates the same bias. The share of the urban areas is TK. 230 and TK. 110 is of the rural areas. It indicates that rural population is neglected comparing to urban population. The share for men in out-patient care is TK. 49.1 and in-patient care is TK. 56.1 and the corresponding figures for women are TK. 43.7 and TK. 60.9 [60]. Overall 17 per cent of the total government health subsidies benefit the poorest quintile of the population, while 25 per cent benefits the richest quintile of the population. The per capita public expenditure for the richest (income quintiles) is TK. 90 (31 per cent) and for the poorest is TK. 39 (13 per cent) for in-patient services. The share for out-patient services is TK. 53 (23 per cent) and Tk. 43 (18 per cent) respectively for these two groups [60]. It indicates that government expenditure pattern is towards urban areas, non-poor and males. The public health services are meant for the economically backward strata of the society but it is pro-rich in practice.

**Table 4:** Expenditure Pattern (Figures in Taka)

**Pattern of expenditure**	**Types**	**Out-patient care **	**In-patient care**	**Total**
Per capita subsidy by location	Urban	79.1	117.8	196.9
	Rural	36.7	40.7	77.4
Per capita subsidy by income quintiles	Rich	52.5	90.1	142.6
	poorest	42.8	38.9	81.6
Per capita health expenditure	Male	49.1	56.1	105.2
	female	43.7	60.9	104.6

Source: [60]

### Manpower shortages and level of corruption

The public health system is facing a problem with manpower shortage. There are only 12,000 physicians working in the public sector and more than 2,000 physicians posts laying vacant in the same sector. It is difficult to provide services with the existing manpower. On the other hand, corruption is rampant in the public health care system of Bangladesh. A study confirms the widespread collection of unofficial fees at various level health facilities is a "common form of rent seeking behaviour in Bangladesh" [61]. The Transparency International found that the health sector is the second most corrupt sector after the police sector [62]. The survey found that 48 per cent admitted to government hospital by alternative methods including 56 per cent paid money, 22 per cent used influence, and 18 per cent sought help from hospital staff [62]. The study shows that doctors are most corrupted followed by hospital staff [62]. An editorial of an English daily commented that Bangladesh experiences show "more than their number, corruption and lack of integrity of the doctors are perhaps, more important factors that explain the poor quality of services at the government run hospitals" [63]. So the shortage of manpower as well as corruption makes the public sector in a bad shape. In addition to the above, Gruen et al., maintains that the public sector has incurred problems of equipment, essential supplies, inadequate facilities, lack of cleanliness, long waiting time, absence or lack of doctors and nurses, inappropriate behaviour by doctors, and lack of confidence in public facilities and staff [64]. Moreover poor management, planning and lack of control make the public sector a defunct system. The public health care system has lost its credibility and people have limited confidence in it. This has resulted in the proliferation of private for-profit oriented health care system.

## Summary

### Health system, human rights and under-development

Though the health system is expanding in terms of health care facilities and manpower, it is far from a comprehensive and integrated health service. The health system has tried to improve the quality of care both at domiciliary and institution level. But the result is far from a satisfactory level and a comprehensive quality development plan has not been made so far. The health system in Bangladesh is biased in favour of the rich and the urban elite in the provision of health care though policies stress to the poor. The government emphasizes the construction of buildings of hospitals rather than providing essential medical facilities, and aims more at curative than preventive aspects. There were little quantitative developments; qualitatively it remained in a dismal state.

Though the prime concern of the government commitment and desire is for better provision of health service for the masses, the practical scenario is not good enough. It is disheartening and gloomy. The present health system has not succeeded to materialize and to realize the vision enunciated in government plans, policies and programmes. It is only able to provide basic services for about forty per cent of the population [37,51], which indicates that the rest are unable to access the health system, and this may be regarded as a violation of human rights. The state has largely failed to respect, fulfil, protect and to promote basic human rights. According to UDHR, ICESCR and the Bangladesh Constitution, it is the obligation of the state to institute policies to address human development, which realise human dignity and thus help to promote human rights. But one will find a dismal picture if he or she tries to contextualize the health system and the development level of the health situation and its relation to human rights. The government has not achieved the goal to facilitate progressive realization of minimum entitlements to the population. The political process and social dynamics allow the poorer, weaker and the disadvantaged groups to access their rights in a very limited way. The State has limited capacity in implementing universally accepted social rights as the state has limited economic and human resources. Moreover, poor governance also jeopardizes government capacity in enhancing social rights. On the other hand, victims are unable to fight against the powerful. There are few organizations which speak peoples' rights but they are still in formative stage and could not succeed in realizing demands. In addition to problems of the health system, there are a lot more problems with allied sectors which can greatly contribute to the improvement of the health status of the population [65]. The poor inputs like the shortage of food, drugs, and health facilities and health personnel are the major causes of the poor health status of the Bangladeshis. There are problems with housing, sanitation, safe water, income, employment, education, and accessibility to services that aggravate the poor health status of the population. However, the constraints could be overcome at least moderately if the government would have a strong political will and determination to solve the problems. The government should remember that the ratifying states have obligations under article 12 of ICSECR regardless of their economic development. The article further states that each party should undertake steps for progressive realization of the rights.

So the responsibility lies with the government for the genuine implementation of the right to health. The government does not have ability to facilitate for advancing the fulfilment of minimum provision of health care to the masses. The *Limburg *principles also implies effective use of available resources. The government also has failed to do so in resource allocation and also proper utilization of the resources. The health sector policy, planning, and action have been poorly implemented. Moreover budgetary allocations, manpower distribution, accessibility, availability, spatial distribution of the health institutions are not homogenous and/or equitable which creates problems to promote the health system as a universal character. The lack of political will, corrupt practices of the officials; unwillingness of the public sector doctors to provide services have made the health system unacceptable. Therefore, the health system has not succeeded to establish as a step forward to development, rather it is merely a case of underdevelopment. The right to development has not been constituted and institutionalized in the provision of health care. So the basics of human rights have not been materialized in the health system of Bangladesh.

### What should be government priority?

In order to provide better health care facilities that the country urgently needs for the majority population, is to implement the policies and programmes enumerated in various government documents. Moreover, the government should take a comprehensive approach to address the human rights and health issues to pursue as a path of development which will help to proceed as a right to development. Necessary initiatives should also be taken to create employment opportunity, income generation, and more production of food and services. Active measures should be taken to reduce disparity between the rural and urban areas, the male and female gap, rich and poor and equity in access to food, health care facilities and other ancillary services. There is a further need to bring out explicit actions of various inter-sectoral cooperation on different development practices, as well as various intra-sectoral efforts. These could be made in the field of health, family planning, drugs, pharmaceuticals, medical education and research, agriculture, food, water supply sanitation, drainage, housing, education rural development, social welfare, women's development to put human rights and health agenda on a path of right to development.

## Competing interests

The author(s) declare that they have no competing interests.

## Pre-publication history

The pre-publication history for this paper can be accessed here:


